# Microstructure Evolution and Mechanical Properties of Al-TiB_2_/TiC In Situ Aluminum-Based Composites during Accumulative Roll Bonding (ARB) Process

**DOI:** 10.3390/ma10020109

**Published:** 2017-01-25

**Authors:** Jinfeng Nie, Fang Wang, Yusheng Li, Yang Cao, Xiangfa Liu, Yonghao Zhao, Yuntian Zhu

**Affiliations:** 1 Nano Structural Materials Center, School of Materials Science and Engineering, Nanjing University of Science and Technology, Nanjing 210094, China; niejinfeng@njust.edu.cn (J.N.); xiaofengn@163.com (F.W.); liyusheng@njust.edu.cn (Y.L.); cao_yang_leo@hotmail.com (Y.C.); ytzhu@ncsu.edu (Y.Z.); 2 Key Laboratory for Liquid–Solid Structural Evolution and Processing of Materials, Ministry of Education, Shandong University, Jinan 250061, China; xfliu@sdu.edu.cn; 3 Department of Materials Science and Engineering, North Carolina State University, Raleigh, NC 27695, USA

**Keywords:** accumulative roll bonding (ARB), Al alloy matrix composites, mechanical properties, microstructure

## Abstract

In this study, a kind of Al-TiB_2_/TiC in situ composite was successfully prepared using the melt reaction method and the accumulative roll-bonding (ARB) technique. The microstructure evolution of the composites with different deformation treatments was characterized using field emission scanning electron microscopy (FESEM) and a transmission electron microscope (TEM). The mechanical properties of the Al-TiB_2_/TiC in situ composite were also studied with tensile and microhardness tests. It was found that the distribution of reinforcement particles becomes more homogenous with an increasing ARB cycle. Meanwhile, the mechanical properties showed great improvement during the ARB process. The ultimate tensile strength (UTS) and microhardness of the composites were increased to 173.1 MPa and 63.3 Hv after two ARB cycles, respectively. Furthermore, the strengthening mechanism of the composite was analyzed based on its fracture morphologies.

## 1. Introduction

Aluminum-based metal matrix composites (AMMCs) reinforced by ceramic particles have received increasing attention due to their high specific strength, good wear resistance, excellent dimensional stability and superior damping capacity [1,2]. A combination of these properties is not available in a conventional material. AMMCs are a very important lightweight structural material and are widely used in some critical components in the automobile and aerospace industries and for other structural applications. The properties of AMMCs depend on the type, size, content, bonding and spatial distribution of ceramic particles [3]. The fabrication process significantly affects these properties of AMMCs. Generally, particle-reinforced aluminum composites are produced using several conventional and specific methods; the liquid metallurgy route is widely preferred owing to its simplicity, low cost, near net shape and mass production [4]. Using liquid metallurgy, particles are either added externally or formed in the molten metal; the latter method is called in situ fabrication [5]. It is known that the particle-reinforced composites synthesized in situ have a clearer interface between the matrix and particle reinforcements, and better interfacial thermodynamic stability, than those synthesized with exogenously formed processes [6].

Fine grain sizes in the matrix, uniform distribution of the reinforcement particles through the matrix and strong bonding of reinforcement particles with the matrix certainly improve the mechanical properties of the composites [7,8]. However, the major drawback of in situ composites is the segregation of the reinforcement particles at the grain boundaries [9,10]. It is difficult to avoid segregation during the formation and solidification of the composite melt. It has been found, however, that severe plastic deformation (SPD) processes such as high-pressure torsion (HPT), equal channel angular pressing (ECAP), and accumulative roll-bonding (ARB), can be used to make the distribution of reinforcement particles more homogenous in the matrix [11,12,13,14,15].

In recent years, ARB has been used to fabricate laminated composite sheets with nanocrystalline and ultrafine-grained structures. ARB consists of roll-bonding cleaned and stacked sheets while reducing their thickness by 50%, cutting them into stacks, and then roll-bonding them again [16,17,18]. A number of studies have investigated the microstructures and mechanical properties of multilayered composites produced by ARB such as Al/Cu, Al/Ni, Al/Mg, Al-SiC, Al-B_4_C, and Al-Al_2_O_3_-B_4_C [13,15,19,20,21,22]. However, few studies of the application of ARB on Al-TiB_2_/TiC in situ composites have been reported. In this work, we fabricated Al-TiB_2_/TiC composites through an in situ melt reaction of Al-Ti and Al-B-C alloys and used ARB to improve the mechanical properties of the resulting composites. TiB_2_ and TiC ceramic particles are outstanding reinforcement particles in Al alloys, offering good thermodynamic stability, high hardness, a high melting point, a high modulus, high corrosion resistance, and low density [23,24,25].

## 2. Experimental Procedure

An Al-8B-2C master alloy supplied by Shandong Al&Mg Melt Technology Co. Ltd. (Jinan, China), Ti sponge (99.8%), and commercial pure Al (99.7%) were used as raw materials for this study. Ti was initially added to Al melts in a medium frequency furnace, then the Al-8B-2C alloy was added to the Al-Ti melts, fabricating an Al-3.6Ti-1.12B-0.28C in situ composite. It was calculated that 3.6 wt % TiB_2_ and 1.4 wt % TiC could be formed in the alloy, which we refer to as Al-5 wt % TiB_2_/TiC in the following. A series of sheets (20 mm in length, and 10 mm in width and thickness) were cut from the ingot of the composites for the rolling deformation and further ARB processes.

**Figure 1:**
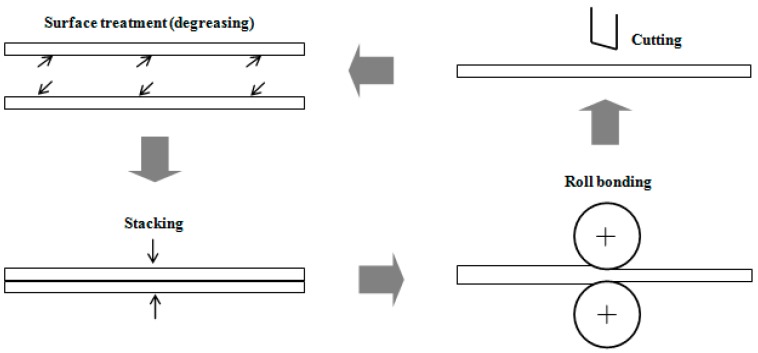
Diagrammatic illustration of the accumulative roll-bonding (ARB) process.

To begin, the as-cast composite sheets were preheated at 300 °C for 3 min in a resistance furnace and rolled on the laboratory rolling mill to a 30% reduction in thickness. The process was repeated three times, obtaining sheets of about 1 mm thickness (a thickness reduction of 90%), which were further processed by ARB. Figure 1 illustrates the principle and procedures of ARB processing. After cleaning and degreasing using acetone, and wire brushing with a circular stainless steel brush to break up thick oxide and create a defined surface roughness of the sheet material, two primary sheets were stacked and fastened together. After preheating at 300°C using the procedure mentioned above, they were roll bonded immediately to a reduction of 50% in thickness in a single cycle. Finally, the roll bonded sheet was cut in half and the same procedures were repeated up to four cycles.

The microstructures of the composites were characterized using a field emission scanning electron microscope (FESEM, Quanta 250F, FEI, Hillsboro, OR, USA) equipped with a energy dispersive X-ray spectrometer (EDS, Oxford Instruments, Oxford, UK), X-ray diffraction (XRD, D8, Bruker, Coventry, Germany) and a transmission electron microscope (TEM, Tecnai20, FEI). The tensile tests were conducted at ambient temperature on the universal test machine (LFM-20, Walter +bai ag, Löhningen, Switzerland) at an initial strain rate of 5.6 × 10^−4^ s^−1^. The tensile test specimens were machined from the processed sheets oriented along the rolling direction (RD) in accordance with the ASTM: E8M standard. Thus, the tensile direction of the specimen was parallel to the RD of the sheets. A Vickers hardness experiment was carried out on the surface RD-TD plane of the multilayered composite using a tester (HMV-G 21DT, Shimadzu, Tokyo, Japan) at a load of 0.49 N held for 15 s. For each specimen, at least seven randomly selected indentations were tested to obtain a mean value with a standard deviation error.

## 3. Results and Discussion

### 3.1. Microstructure Evolution of the Al-5 wt % TiB_2_/TiC Composites

**Figure 2:**
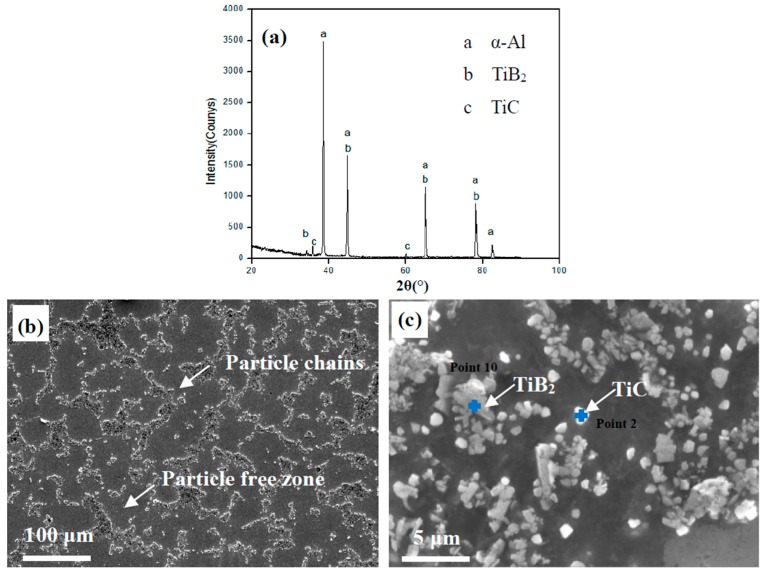

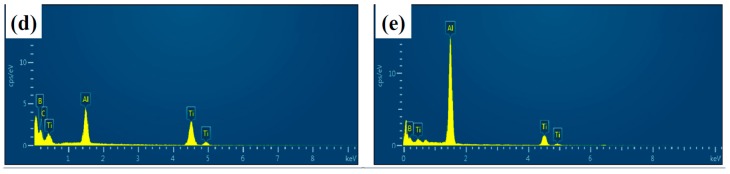
XRD pattern and FESEM micrographs of Al-TiB_2_/TiC in situ composites: (**a**) XRD pattern; (**b**,**c**) Microstructures at low and high magnification; (**d**,**e**) EDS point analysis for the particles.

The XRD pattern of the prepared Al-5 wt % TiB_2_/TiC in situ composites is shown in Figure 2a. The XRD pattern shows that TiB_2_ and TiC phases were formed in these composites. Figure 2b shows the microstructures of the composites, and many reinforcement particles can be observed in the matrix. Notice that most of the reinforcement particles are distributed along the grain boundaries, and the particle-agglomerated clusters formed long particle chains. Meanwhile, large particle-free zones formed in the interior of the grains. In the magnified image (Figure 2c), the white particles are TiC and the gray particles are TiB_2_ [26]. EDS experiments were also used to identify TiC and TiB_2_ particles, as shown in Figure 2d,e. The TiB_2_ and TiC particles were in the range of 0.5–2 μm, and the small-sized particles easily formed large agglomerated clusters due to their large surface-to-volume ratio and their attractive Van der Waals interactions [27].

**Figure 3:**
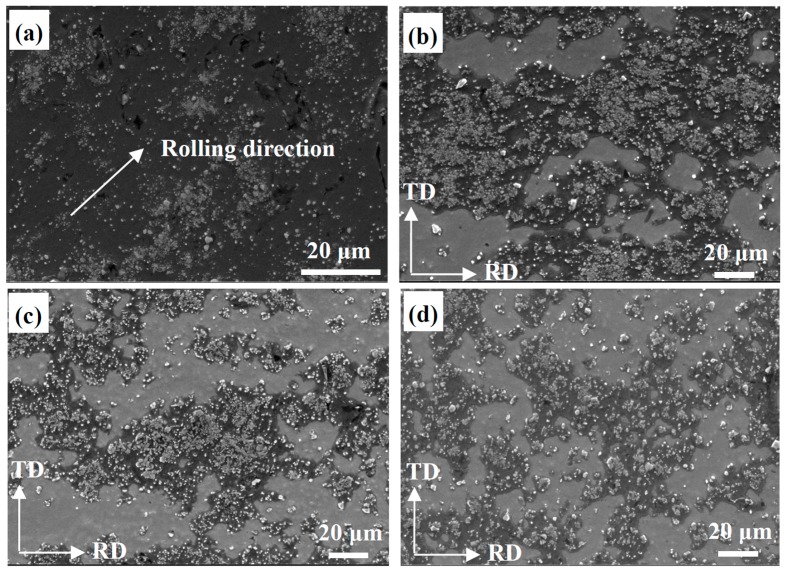
Microstructures ofAl-TiB_2_/TiC in situ composites with different deformation processes (in RD-TD plane). (**a**) Rolling for 90% reduction; (**b**–**d**) Following one, two and four ARB cycles, respectively.

In order to make the distribution of TiC and TiB_2_ reinforcement particles more uniform in the Al matrix, the composites was processed by rolling deformation after preheating. Figure 3a shows the microstructures of rolled Al-5 wt % TiB_2_/TiC composites after reducing their thickness by 90%; it can be seen that the size of the particle-free zone and the amount of agglomerated reinforcement particles became smaller. The composites after rolling for a 90% reduction were further processed using ARB. Figure 3b–d show the microstructures (in the RD-TD planes) of the composites with 90% reduction rolling in combination with one, two, and four ARB cycles, respectively. The distribution of the reinforcement particles in the matrix is more homogenous after ARB treatment than in the as-cast one, shown in Figure 2b. After the fourth cycle, the particle-free zone in the matrix became smaller and the particle distribution in the matrix became more uniform.

**Figure 4:**
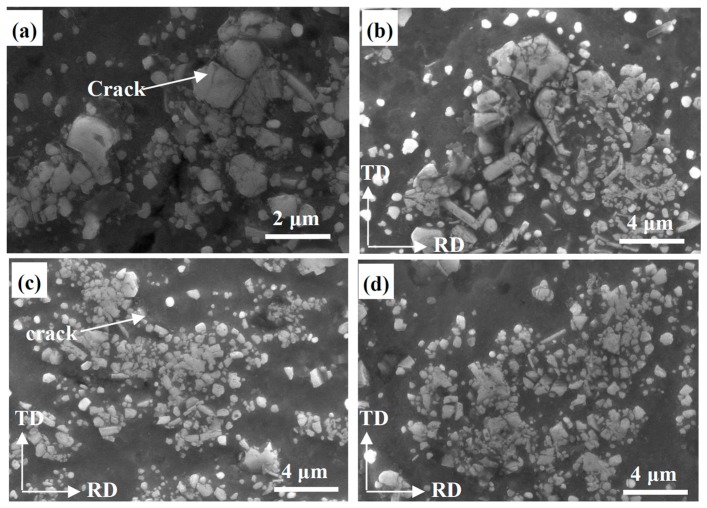
Effect of deformation on the particle distribution of the composites in the RD-TD plane. (**a**) Rolling for 90% reduction; (**b**–**d**) Following one, two and four ARB cycles, respectively.

Figure 4 demonstrates the SEM micrographs of the microstructures of Al-5 wt % TiB_2_/TiC composites after different deformation processes at a higher magnification. As shown in Figure 4a, some cracks appeared in the large close-packed particle clusters, formed by plastic deformation during rolling. We suggest that the large particles are actually composed of multiple smaller ones, due to their attractive Van der Waals interactions reducing the total surface energy in the melt. With increased deformation after multiple ARB cycles (Figure 4b,c), the large agglomerated particle cracked and was divided into several submicro-sized ones. By increasing the ARB processing up to four cycles, the number of small particles of submicro size was increased, due to the separation of the particle clusters, and almost all the resulting particles were smaller than 1 μm. Furthermore, the distribution of reinforcement particles in the matrix became more homogenous, as shown in Figure 4d.

**Figure 5:**
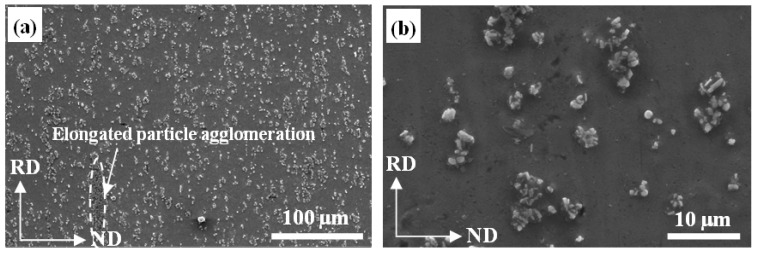

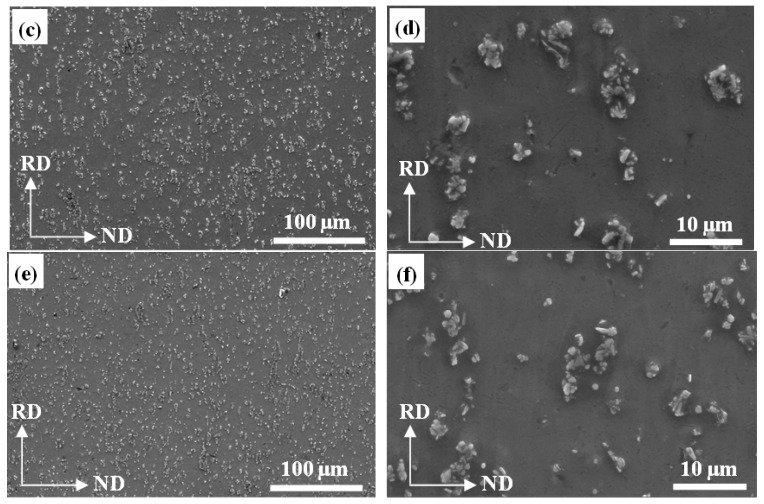
Microstructure evolution of Al-TiB_2_/TiC in situ composites with increased ARB passes in RD-ND plane: (**a**,**b**) One cycle; (**c**,**d**) Two cycles; (**e**,**f**) Four cycles.

The microstructures of the Al-5 wt % TiB_2_/TiC composites following increasing ARB cycles in the RD-ND plane were also investigated, and are shown in Figure 5. After the first cycle (Figure 5a,b), the particle chains in the original sample almost disappeared and a few elongated agglomerated particle clusters existed along the rolling direction. In the magnified image in Figure 5b, it can be seen that the reinforcement particle clusters were broken by the stress of rolling and most of them separated from each other. With increasing ARB cycles, the distribution of particles in the matrix became much more uniform as shown by Figure 5c,d. As the ARB process progressed up to four cycles, a microstructure with uniformly distributed TiB_2_ and TiC reinforcement particles was obtained (Figure 5e,f). This phenomenon is attributed to the high deformability of the Al matrix under the stress of rolling. Furthermore, compared with Figure 3 and Figure 4, the microstructure shows that the uniformity of the RD-ND plane was much better that of the RD-TD plane [28].

### 3.2. Grain Structures of the Al-5 wt % TiB_2_/TiC Composites

**Figure 6:**
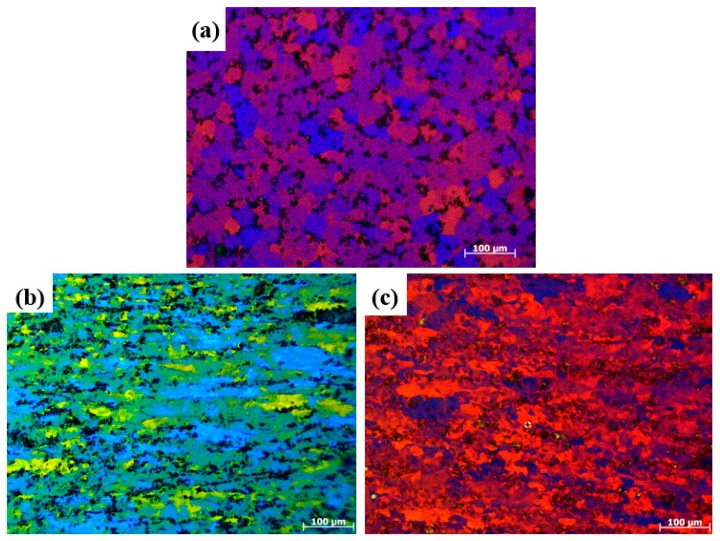
Polarized light optical micrograph of Al-TiB_2_/TiC in situ composites: (**a**) As cast; (**b**) Rolled for 90% reduction; (**c**) After one ARB cycle.

The grain structures of the composites were shown by the polarized light optical micrograph after electroetching the polished specimens. The as-cast Al-TiB_2_/TiC composite has an average grain size of 30.6 μm, as shown in Figure 6a. After rolling for 90% reduction, the grains of the Al matrix were elongated and many grains were refined significantly due to the fragmentation of the elongated grains during the rolling process, as shown in Figure 6b. With ARB processing, the Al grains were further refined after one cycle, as illustrated in Figure 6c.

**Figure 7:**
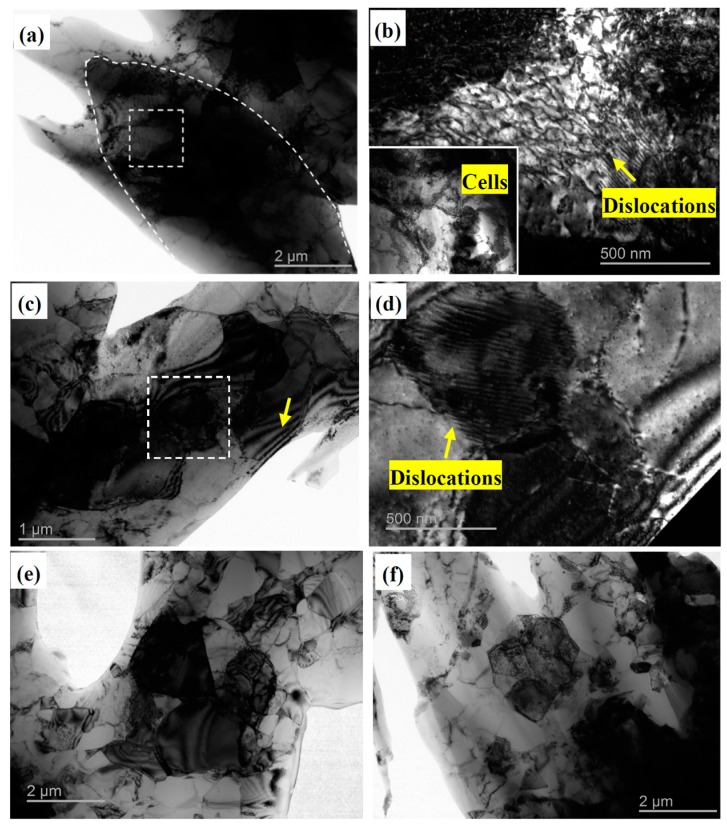
Typical TEM micrographs of Al-5 wt % TiB_2_/TiC composites: (**a**,**b**) After rolling for 90% reduction; (**c**,**d**) After one ARB cycle; (**e**) After two ARB cycles; (**f**) After four ARB cycles.

The TEM micrographs of the Al-TiB_2_/TiC composites with different deformation histories are shown in Figure 7. Figure 7a,b give the microstructures of the Al-TiB_2_/TiC composite after its thickness was reduced by 90% by rolling: the matrix Al grains were elongated and many dislocation cells were formed. Furthermore, a number of dislocation lines can be clearly seen at the higher-magnification dark field image shown in Figure 7b. After treatment by one ARB cycle, grain size decreased and the number of dislocation tangles in the grains also decreased, as shown in Figure 7c. The most prominent microstructural change after one ARB cycle was a drastic decrease in the dislocation density when compared with the 90%-reduced rolled sample. The dislocation cell size also became finer (Figure 7d), suggesting dislocation rearrangement during the ARB treatment. Moreover, contours can be seen mostly near the boundaries, reflecting the internal stress existing in the grains.

For the microstructures after deformation by two ARB cycles, shown in Figure 7e, an obvious decrease of grain size was observed, and the interior of most grains was very clean. The boundaries of most grains were very sharp, suggesting the misorientation angles between the grains were increased and high angle boundaries were formed. The dislocation density inside the grains was very low in comparison with that observed after one ARB cycle, indicating that the annihilation of dislocations inside the grains occurred, which is in accordance with the fact that the development of fine structures during SPD is accompanied by a decrease in dislocation density at large strains. After four ARB cycles, more grains were refined and some ultrafine equiaxed grains were formed, as shown in Figure 7f.

Generally, the formation of ultrafine structures in the highly strained materials was regionally inhomogeneous because of the friction between the rolls and the strip surfaces [29]. The grain refinement mechanism during the SPD process has been explored extensively by researchers [30], who have found that fine dislocation cell structures with low-angle grain boundaries are first created firstly at a small strain, after which the cell structures become finer and their misorientation angle increases [31,32]. Finally, a fine grain structure with distinguished high angle boundaries is generated at a higher plastic strain.

### 3.3. Mechanical Properties of the In Situ Al-5 wt % TiB_2_/TiC Composites

**Figure 8:**
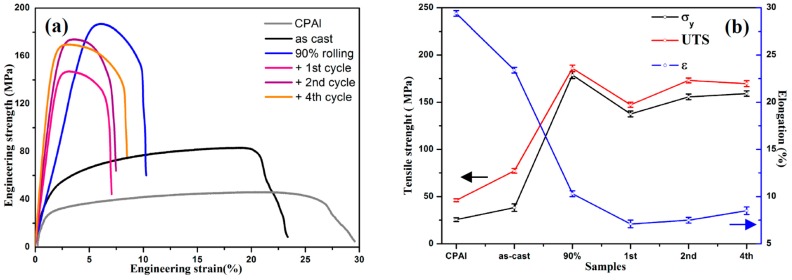
Tensile properties of the Al-TiB_2_/TiC composites. (**a**) Typical engineering stress-strain curves of the composites and CPAl; (**b**) Variations of σ_y_, UTS and elongation of the composites as a function of different deformation treatments.

The typical engineering stress-strain curves of different Al-TiB_2_/TiC composites and commercial pure Al (CPAl) are shown in Figure 8a. Compared with commercial pure Al, the tensile strength of the in situ Al-TiB_2_/TiC composite was enhanced greatly by the reinforcement particles. Meanwhile, the composites with 90% reduction rolling and after subsequent ARB cycles showed higher strength and lower ductility compared to the as-cast one. The composites’ variation of yield strength (σ_y_), ultimate tensile strength (UTS), and elongation to failure (ε) under different deformation treatments, obtained from the engineering stress-strain curves, are presented in Figure 8b.

Compared with those of the as-cast composite, the σ_y_ and UTS of the composites after rolling for 90% reduction were significantly increased, from 38.5 and 77.2 MPa to 182.8 and 185.9 MPa, respectively. However, ductility had a dramatic decrease. After further treatment with one ARB cycle, both the σ_y_ and UTS of the composite were decreased, to 137.6 and 147.3 MPa, respectively. They were slightly increased to 155.7 and 173.1 MPa after the second ARB cycle. After the fourth ARB cycle, σ_y_ was almost unchanged and the UTS deceased slightly. Generally, the elongation of the composites decreased dramatically after the first ARB treatment, was slightly increased with further ARB cycles, and exceeded 8.5% after the fourth ARB cycle treatment.

Furthermore, the microhardness of the Al-TiB_2_/TiC composites after rolling and subsequent ARB cycles was also measured on the surface of RD-ND planes, and is shown in Table 1. The variations in hardness were similarto those of the tensile strength. The hardness of the composite rapidly increased after rolling and the initial ARB cycles, showing a lower rate of increase after further ARB treatment. After 90% reduction rolling, the composite’s hardness was 59.9 Hv, 35.5% higher than that of the as-cast composite, 44.2 Hv. It reached its maximum value of 63.3 Hv after the second ARB cycle, and decreased slightly after the fourth ARB cycle.

It is known that strain hardening or dislocation strengthening plays the main role in the composite’s increased strength after the initial rolling and ARB cycles [33,34,35]. After 90% reduction rolling, a high density of dislocations was formed in the grains, as shown in Figure 7a,b. Therefore, the increase in the σ_y_ and UTS for the composites after 90% reduction rolling could be attributed to the increased dislocation density (strain hardening). Meanwhile, work hardening leads to a quick decrease of the ductility of the composites. However, with increased strain through one ARB cycle, more dislocations were annihilated and the dislocation density decreased in the grains, leading to the decrease of the σ_y_ and UTS of the composites. Once the dislocation density saturates due to the dynamic balance between dislocation generation during plastic deformation and dislocation annihilation through recovery processes, strength is determined by the grain size. After the second ARB cycle treatment, the grain size was obviously refined in comparison to that after one ARB cycle treatment, leading to increased σ_y_ and UTS. In addition, the microstructures shown in Figure 3 and Figure 5 indicate that with increasing ARB cycles, the distribution of the reinforcement particles in the aluminum matrix became more uniform and the average size of the particles was reduced to less than 1.0 μm. Thus more particles became suitable reinforcements for Orowan strengthening [33], which can also contribute to increased tensile strength and ductility [7,8,15,34]. In the same way deformation promotes grain refinement, we suppose that a high temperature could promote boundary mobility at higher strains. Therefore, we suggest that the matrix grain size could slightly increase after the fourth ARB cycle, leading to a slight decrease in the UTS and an increase in elongation. In addition, we suspect microcracks formed in the samples at higher strain, which can also decrease the UTS of the composites.

**Figure 9:**
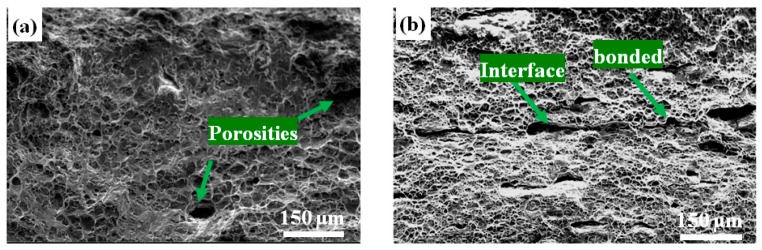

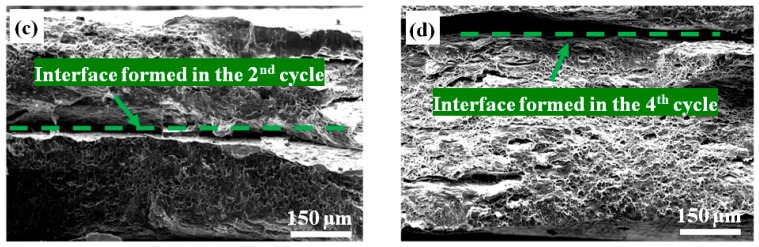
Tensile fracture surfaces of theAl-TiB_2_/TiC composites: (**a**) As cast; (**b**–**d**) After one, two and four ARB cycles, respectively.

Figure 9 shows the fracture surfaces of the Al-TiB_2_/TiC composites after tensile tests were performed to investigate the failure mechanisms and bonding characteristics of the composites. A typical ductile fracture with a large number of dimples and shear zones can be seen in the as-cast Al-TiB_2_/TiC composite (Figure 9a); porosities (indicated by arrows) were also found. After the first ARB cycle, the size of the dimples at the fracture surface was much smaller, as shown by Figure 9b. Meanwhile, it can be seen that during the tensile test for the composites, debonding started in the interface between the two layers formed during the first ARB cycle (Figure 9b). Note that some areas at the interface were also bonded tightly with no cracks, indicating that the two layers were well bonded. It is known that new interfaces form continuously during the ARB process. With increasing cycles, as shown in Figure 9c, total debonding between layers occurred during the tensile test, and in the second cycle, microcracks developed at the newer interface, while the interface formed in the first cycle cannot be seen clearly. The interfaces formed in the first cycle were bonded together tightly in the second cycle, and good bonding between the composite layers can be obtained. By increasing to the fourth cycle, the composite sheet consisted of 16 layers, and only one separated interface could be seen on the fracture surfaces, which are similar with that seen in Figure 9c. In fact, total separation between layers happened at the newest interface formed in the fourth cycle, as shown in Figure 9d. Therefore, we consider that separation in the last cycle will decrease the strength of the composites to some extent. We also assume that further rolling can be applied to the fourth ARB cycle sample to improve the bonding between layers.

**Figure 10:**
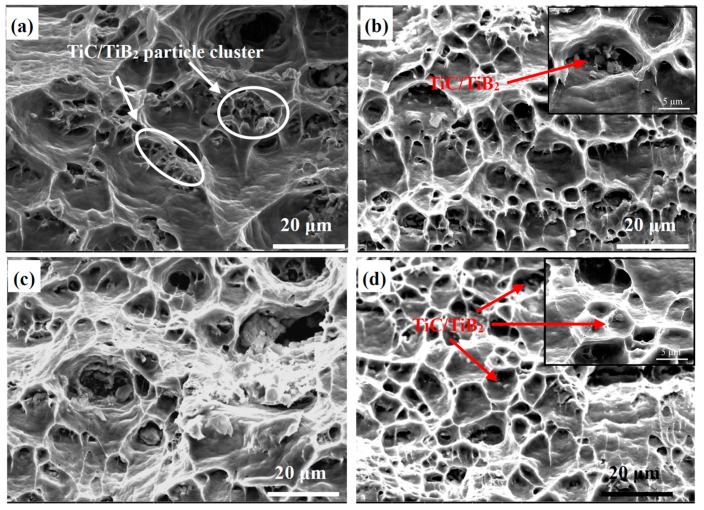
Fractography of Al-TiB_2_/TiC composites at higher magnification: (**a**) As cast; (**b**–**d**) After one, two and four ARB cycles, respectively.

The fracture surfaces at higher magnification are shown in Figure 10. The as-cast composite exhibits a typical ductile facture with deep equiaxed dimples, and most of the reinforcement particles are distributed in the dimples (Figure 10a). It is known that ductile fracture occurs by the nucleation of microvoids, followed by their growth and coalescence [36]. The presence of particles at the bottom of some dimples can be considered as evidence for the nucleation of voids from the particle-matrix interfaces [37]. After one ARB cycle, the size of the dimples decreased significantly, as shown in Figure 10b, and the dimple size decreased slightly more with increased ARB cycles. It has been found that the presence of the particles can change the morphology of dimples so that the dimples nucleated at the particle sites are deeper and larger than those nucleated in other regions. As shown in Figure 5, the reinforcement particles were uniformly distributed in the matrix after four ARB cycles. Therefore, shallow and small dimples were found at the fracture surfaces of the composites treated by more ARB processes (Figure 10b–d).

## 4. Conclusions

In summary, Al-TiB_2_/TiC composites were fabricated by an in situ melt reaction and subsequent rolling and ARB processes. Our main conclusions are:

(1) The in situ TiC and TiB_2_ were formed by the reaction of Al-8B-2C and Al-Ti melts. The TiB_2_ and TiC particles, ranging from 0.5 to 2 μm in size, agglomerated along the grain boundary in the as-cast composites.
(2) The large particle agglomerations in the composites were effectively reduced after subsequent rolling and ARB processes. The distribution of TiB_2_ and TiC reinforcement particles in the matrix became more uniform with increased ARB cycles.
(3) The mechanical properties of the Al-TiB_2_/TiC in situ composites were significantly improved by the ARB process. The UTS and microhardness of the composites were increased to 173.1 MPa and 63.3 Hv, respectively, after the second ARB cycles. However, ductility was dramatically decreased, from 23.4% to 8.5%.
(4) The fracture surface of Al-TiB_2_/TiC composites showed many dimples, indicating ductile-type fracture. Most of the TiB_2_ and TiC reinforcement particles were located in the centers of the dimples.

## Figures and Tables

**Table 1:** The average hardness of Al-TiB_2_/TiC composites after rolling and different ARB cycles.

Samples	Hardness/Hv
as cast	44.2
90%	59.9
1st	56.6
2nd	63.3
4th	61.8
